# Association between pregabalin use and delirium in critically ill patients aged 60 and older: a retrospective analysis of the MIMIC-IV database

**DOI:** 10.1515/med-2025-1354

**Published:** 2026-01-22

**Authors:** Jijie Xiao, Sisi Qin, Shiqi Yuan, Yang Liu, Yi Wang, Ningjun Li, Li Kou

**Affiliations:** Department of Neurology, The Fifth Affiliated Hospital of Sun Yat-Sen University, Zhuhai, China; Department of Radiology, The Third Affiliated Hospital of Southern Medical University, Guangzhou, China; Department of Critical Care Medicine, The Fifth Affiliated Hospital of Sun Yat-Sen University, Zhuhai, China; Neurology Ward 1, Jinyang Hospital Affiliated to Guizhou Medical University, Guiyang, China

**Keywords:** pregabalin, delirium, elderly, critical care, MIMIC-IV

## Abstract

**Objectives:**

Delirium is a frequent complication in elderly and critically ill patients, associated with significant morbidity and mortality, prolonged hospitalization, increased healthcare costs, and long-term cognitive impairment, emphasizing the need for early identification and prevention strategies. In this study, we aimed to assess the association between pregabalin use and the risk of delirium in elderly intensive care unit (ICU) patients.

**Methods:**

This retrospective cohort study analyzed data from the Medical Information Mart for Intensive Care IV (MIMIC-IV) database. Propensity score matching (PSM) was applied to control for baseline confounders. Multivariate logistic regression, inverse probability of treatment weighting (IPTW), and subgroup analyses were conducted to validate the findings.

**Results:**

Out of 12,327 patients, 309 pregabalin users were matched with 1,236 non-users through 1:4 PSM. Pregabalin use was significantly associated with increased delirium risk in patients aged 60 and older, with odds ratios (ORs) of 1.72 (1.31–2.25), 1.90 (1.44–2.49), 1.89 (1.37–2.58), and 1.91 (1.58–2.31) across models. Subgroup analyses confirmed this association across most variables. Notably, the risk was significantly higher among patients not receiving analgesia (p for interaction <0.05).

**Conclusions:**

Pregabalin use is associated with an elevated risk of delirium in critically ill patients aged 60 and above. These findings have important implications for medication management and delirium prevention in the ICU.

## Introduction

Delirium is an acute neurocognitive disorder characterized by impairments in attention, awareness, and cognition. It is associated with substantial morbidity and mortality, prolonged hospitalization, increased healthcare costs, and long-term cognitive decline [1], [2], [3]. Predominantly affecting the elderly, delirium often arises from acute medical conditions [4], 5] and represents the most common manifestation of cerebral dysfunction in critically ill patients [6]. Its prevalence in intensive care units (ICUs) can reach up to 87 %, particularly among patients with burns, those undergoing emergency surgery, or individuals requiring mechanical ventilation [7], 8]. Major risk factors include advanced age, baseline cognitive impairment [9], frailty, multiple comorbidities, psychiatric conditions such as depression [10], 11], alcohol use, malnutrition [12], acute illness, certain medications (e.g., opioids, sedatives, anticholinergics) [7]., substance withdrawal, trauma, surgery, and neurological conditions like stroke [13]. For instance, sepsis may trigger delirium through mechanisms involving neuroinflammation, impaired cerebral perfusion, blood-brain barrier (BBB) disruption, and altered neurotransmission [14]. Given the limited effectiveness of current treatments, managing delirium in the ICU remains challenging. Thus, early prevention and detection – through cognitive screening tools and modification of risk factors – are critical.

Pregabalin, a gamma-aminobutyric acid (GABA) analogue, is approved for the treatment of fibromyalgia, neuropathic pain, generalized anxiety disorder, and epilepsy [15]. It is commonly used as adjunct therapy for partial seizures, diabetic neuropathy, postherpetic neuralgia, fibromyalgia, and spinal cord injury-related pain [16], 17]. Adverse effects include dizziness, somnolence, angioedema, and, rarely, rhabdomyolysis [15], 18]. Neuropsychiatric side effects such as dizziness, drowsiness, fatigue, confusion, hallucinations, agitation, and aggression occur in approximately 35.2 % of users [19], 20]. However, despite the high incidence of delirium in ICU settings, its potential link to pregabalin remains largely unrecognized, with only a few case reports documenting pregabalin-induced delirium [21], [22], [23].

This study aimed to examine the association between pregabalin use and the incidence of delirium in elderly ICU patients, and to explore potential underlying mechanisms. Propensity score matching (PSM) was employed to control for confounding variables, enabling a more accurate comparison between patients exposed to pregabalin and those unexposed.

## Methods

### Data source

This retrospective study utilized data from the Medical Information Mart for Intensive Care IV (MIMIC-IV, version 2.0) database, which containing de-identified patient records from Beth Israel Deaconess Medical Center ICUs (2008–2019). Approval was obtained from the institutional review boards of both Beth Israel Deaconess Medical Center (Boston, MA, USA) and the Massachusetts Institute of Technology (Cambridge, MA, USA). Informed consent was waived due to data anonymization. One author (Sisi Qin) completed the required CITI training (ID: 51305476). Because this study involved only secondary analysis of fully de-identified data from the MIMIC-IV database, no additional ethical approval was required.

### Participant selection

All patients were identified from the MIMIC-IV database (version 2.0). Inclusion criteria comprised: (1) individuals aged ≥60 years admitted to the ICU for the first time, and (2) an ICU stay of at least two days. Exclusion criteria encompassed: (1) a diagnosis of dementia, (2) mild cognitive impairment (MCI), (3) epilepsy, and (4) absence of a Confusion Assessment Method for the Intensive Care Unit (CAM-ICU) assessment. A total of 12,327 patients met the criteria and were included in the final analysis.

### Data extraction and outcome measures

Raw data were extracted within the first 24 h of ICU admission using Structured Query Language (SQL) via PostgreSQL (version 10.17). Extracted included age, gender, hypertension, diabetes, heart failure, respiratory failure, sepsis, surgery, mechanical ventilation, analgesia, sedation, systolic blood pressure (SBP), diastolic blood pressure (DBP), white blood cell count (WBC), platelet count (PLT), creatinine (Cr), sodium, glucose, Simplified Acute Physiology Score II (SAPS II), Glasgow Coma Scale (GCS), ICU length of stay (ICU Los), and occurrence of delirium. Variables with >20 % missing data were excluded to reduce bias. For those with less than 20 % missingness, multiple imputation was conducted using the “mice” package in R, ensuring that all retained variables remained below the threshold. Likelihood-based model selection methods, including Akaike’s Information Criterion (AIC) and Bayesian Information Criterion (BIC), were applied to identify the most suitable imputation model [24].

Patients with documented pregabalin use before or after ICU admission were classified as pregabalin-exposed. Delirium was defined as at least one positive assessment using the Confusion Assessment Method for the Intensive Care Unit (CAM-ICU) [25] during the ICU stay. The CAM-ICU assessments were performed by trained ICU nurses as part of routine clinical practice, and the results are documented in the MIMIC-IV database. For the primary analysis, the timing of delirium onset was defined as the datetime of the first positive CAM-ICU assessment. To establish a clear temporal sequence supporting causal inference, we required that the first documented pregabalin administration must have occurred prior tothe first positive CAM-ICU assessment for a patient to be considered as having an exposure-related outcome. The cumulative pregabalin dose during the ICU stay was also calculated. The primary outcome was the incidence of ICU delirium. No significant collinearity was detected among covariates (Supplementary Figure S1 and S2).

### Statistical analysis

Patients were categorized into pregabalin-exposed and non-exposed groups. Continuous variables are presented as mean±SD or median (IQR), and categorical variables as proportions. Group comparisons used ANOVA, Kruskal-Wallis, or Chi-squared tests.

Propensity score matching (PSM) was performed (1:4 ratio, caliper=0.1) using the ‘MatchIt’ package in R (version 4.4.1) to balance covariates (e.g., age, comorbidities, scores). Balance was assessed using standardized mean differences (SMD). The association between pregabalin and delirium was evaluated using multivariable logistic regression, reported as odds ratios (OR) with 95 % confidence intervals (CI). Inverse probability of treatment weighting (IPTW) and subgroup analyses (e.g., by age, comorbidities) were also conducted, with interactions tested via likelihood ratio tests, with statistical significance defined as p<0.05.

## Results

### Baseline characteristics before and after PSM

**Figure 1:**
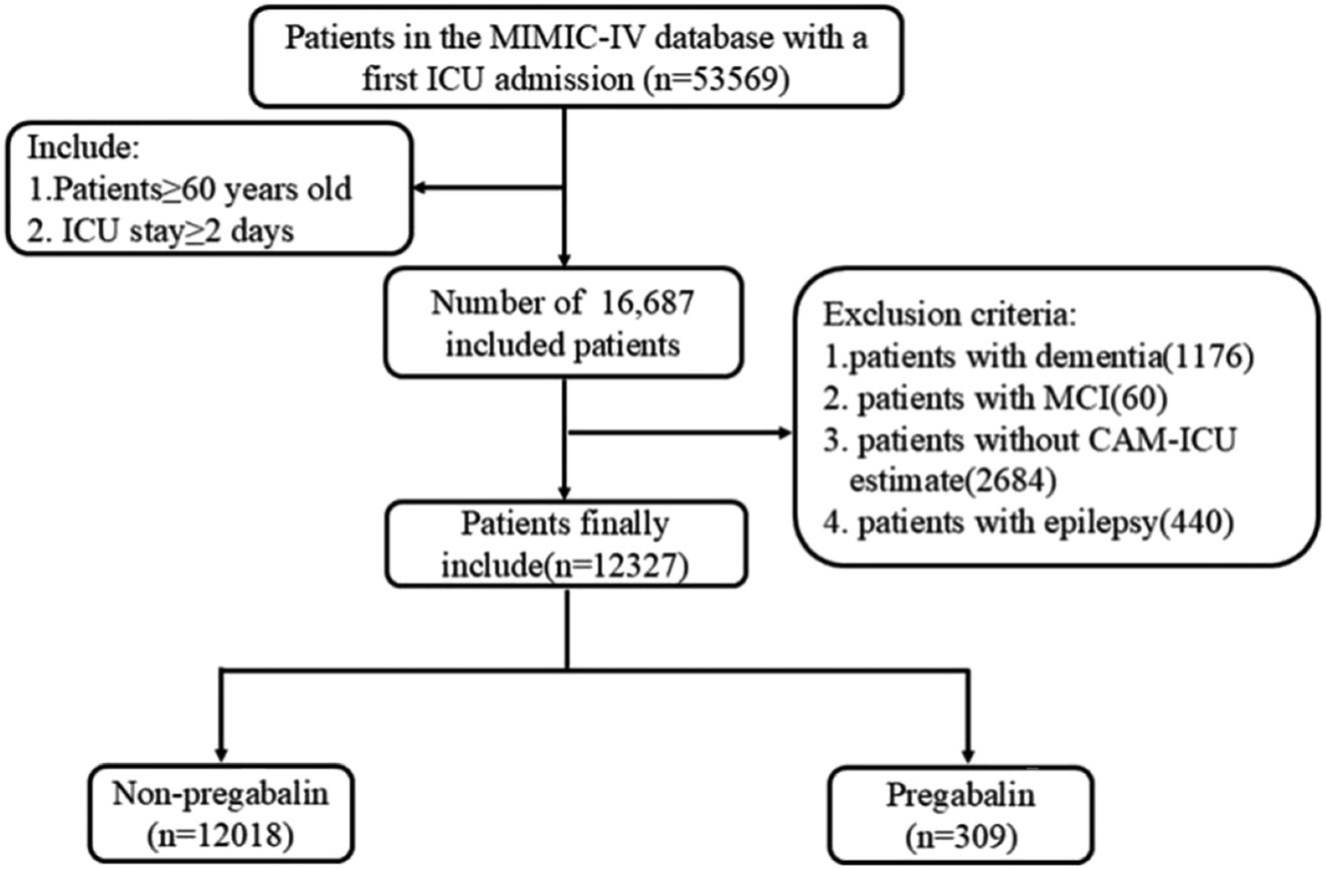
Patient flow throughout the trial.

**Figure 2:**
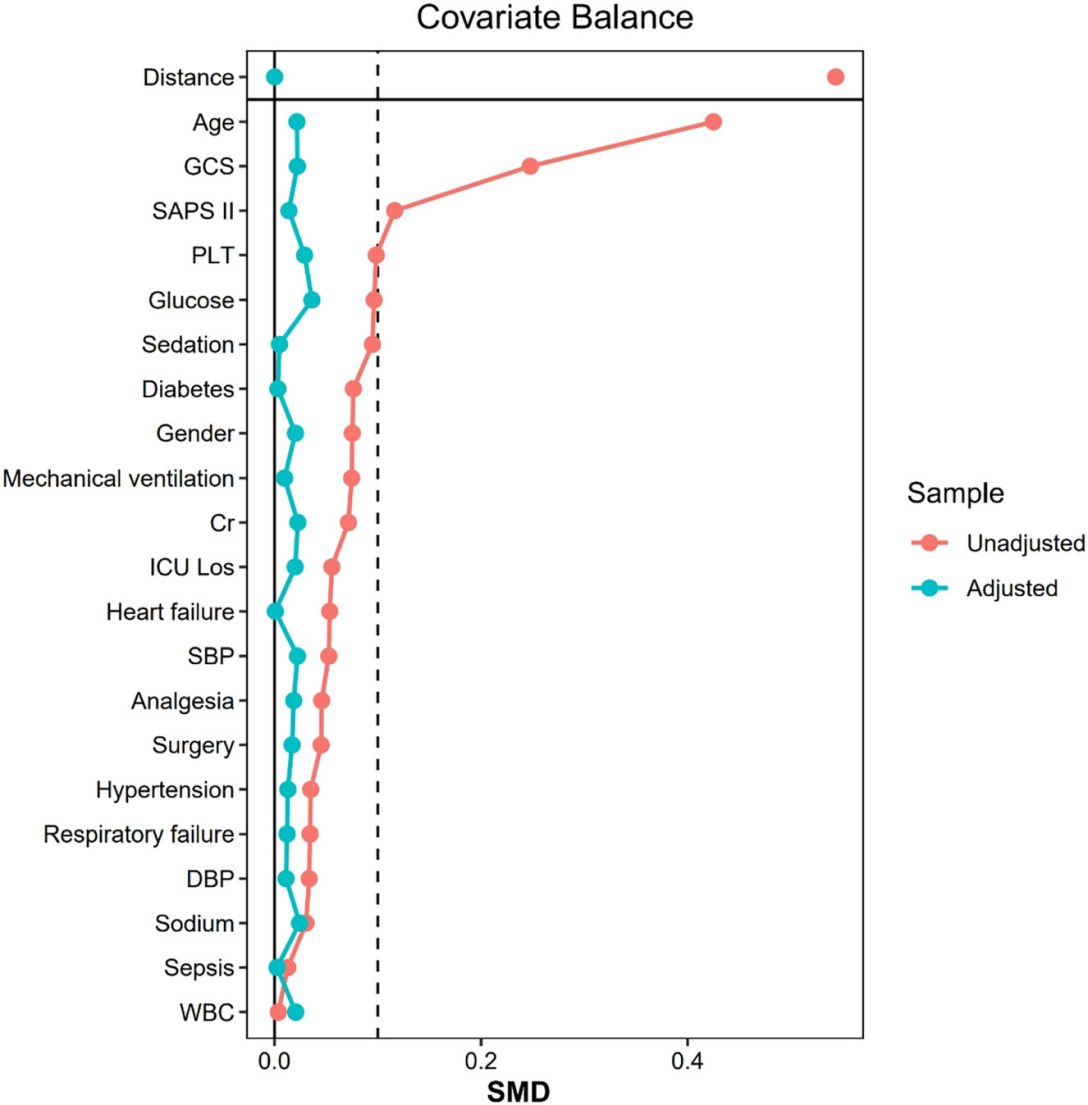
SMD of variables before and after PSM.

A total of 12,327 patients were included in the analysis, with 309 receiving pregabalin. The patient selection flow is presented in Figure 1, and baseline characteristics before and after PSM are summarized in Table 1. Before matching, pregabalin users had a higher proportion of females and diabetes, but lower rates of surgery, mechanical ventilation, and sedation (all p<0.05; Table 1). After 1:4 PSM (309 exposed vs. 1,236 controls), all covariates were balanced (SMD<10 %, p>0.05; Figure 2).

**Table 1:** Baseline characteristics stratified by pregabalin use before and after PSM.

Before PSM					After PSM			
Characteristic	Non-pregabalin n=12,018	Pregabalin n=309	SMD	p-Value	Non-pregabalin n=1,236	Pregabalin n=309	SMD	p-Value
Age	74 (67, 82)	70 (65, 77)	0.40	<0.001	70 (65, 76)	70 (65, 77)	0.02	0.799
Gender			0.15	0.010			0.04	0.567
F	5,356 (45 %)	161 (52 %)			619 (50 %)	161 (52 %)		
M	6,662 (55 %)	148 (48 %)			617 (50 %)	148 (48 %)		
Hypertension	6,488 (54 %)	156 (50 %)	0.07	0.246	608 (49 %)	156 (50 %)	0.03	0.731
Diabetes	4,022 (33 %)	127 (41 %)	0.16	0.006	504 (41 %)	127 (41 %)	0.01	0.969
Heart failure	4,104 (34 %)	89 (29 %)	0.12	0.058	355 (29 %)	89 (29 %)	0.00	1.000
Respiratory failure	3,826 (32 %)	109 (35 %)	0.07	0.223	421 (34 %)	109 (35 %)	0.03	0.738
Sepsis	2,179 (18 %)	60 (19 %)	0.03	0.614	243 (20 %)	60 (19 %)	0.01	0.987
Surgery	1,359 (11 %)	21 (6.8 %)	0.16	0.017	105 (8.5 %)	21 (6.8 %)	0.06	0.390
Mechanical ventilation	5,409 (45 %)	116 (38 %)	0.15	0.011	476 (39 %)	116 (38 %)	0.02	0.804
Analgesia	5,177 (43 %)	119 (39 %)	0.09	0.123	453 (37 %)	119 (39 %)	0.04	0.589
Sedation	6,973 (58 %)	150 (49 %)	0.19	0.001	606 (49 %)	150 (49 %)	0.01	0.929
SBP	121 (104, 139)	118 (104, 139)	0.05	0.411	118 (102, 137)	118 (104, 139)	0.02	0.650
DBP	65 (54, 77)	65 (54, 78)	0.04	0.895	66 (56, 77)	65 (54, 78)	0.01	0.297
WBC	11.0 (8.0, 15.1)	11.6 (8.4, 15.3)	0.00	0.209	11 (8, 16)	12 (8, 15)	0.02	0.405
PLT	182 (132, 244)	198 (143, 263)	0.10	0.008	194 (140, 256)	198 (143, 263)	0.03	0.591
Cr	1.00 (0.70, 1.50)	0.90 (0.70, 1.40)	0.07	0.044	0.90 (0.70, 1.30)	0.90 (0.70, 1.40)	0.02	0.994
Sodium	139 (136, 141)	139 (136, 141)	0.03	0.796	139 (136, 141)	139 (136, 141)	0.02	0.766
Glucose	130 (108, 165)	133 (109, 174)	0.11	0.078	131 (106, 174)	133 (109, 174)	0.04	0.385
SAPS II	39 (32, 47)	36 (31, 46)	0.12	0.004	37 (30, 45)	36 (31, 46)	0.01	0.765
GCS	14 (10, 14)	14 (12, 15)	0.23	0.002	14 (12, 15)	14 (12, 15)	0.02	0.440
ICU Los	3.7 (2.7, 6.1)	3.9 (2.6, 6.0)	0.05	0.930	3.6 (2.6, 5.9)	3.9 (2.6, 6.0)	0.02	0.556
Outcome variable								
Delirium	1,776 (15 %)	71 (23 %)			169 (14 %)	71 (23 %)		

### Association between pregabalin use and delirium risk in elderly ICU patients

Multivariate logistic regression was conducted to assess the association between pregabalin use and the risk of delirium in elderly ICU patients. As shown in Table 2, pregabalin use was significantly associated with an increased risk of delirium in the unadjusted model (OR: 1.72; 95 % CI: 1.31–2.25; p<0.001). After adjustment for confounding variables via stepwise regression, the association remained significant and slightly stronger (OR: 1.90; 95 % CI: 1.44–2.49; p<0.001). This relationship was further supported by post-matching analyses using PSM (OR:1.89, 95 % CI:1.37–2.58, p<0.001) and IPTW (OR:1.91, 95 % CI:1.58–2.31, p<0.001). Notably, the majority of pregabalin-exposed patients received their first dose within 24 h of ICU admission, and the median cumulative dose was 300 mg (IQR: 150–450) over the ICU stay.

**Table 2:** Relationship between pregabalin use and delirium risk in ICU patients aged 60 and above.

Analysis	No-pregabalin	Pregabalin	
		OR (95%CI)	p-Value
Unadjusted	Ref	1.72 (1.31, 2.25)	<0.001
Multivariable adjusted	Ref	1.90 (1.44, 2.49)	<0.001
PSM	Ref	1.89 (1.37, 2.58)	<0.001
IPTW	Ref	1.91 (1.58, 2.31)	<0.001

Unadjusted: without adjustment. Multivariable adjusted: adjusted for all baseline variables using the optimal model selected via stepwise regression. PSM: propensity score matching, adjusted for age, gender, hypertension, diabetes, heart failure, respiratory failure, sepsis, surgery, mechanical ventilation, analgesia, sedation, SBP, DBP, WBC, PLT, Cr, sodium, glucose, SAPS II, GCS, and ICU, Los. IPTW: inverse probability of treatment weighting.

### Subgroup analyses

**Figure 3:**
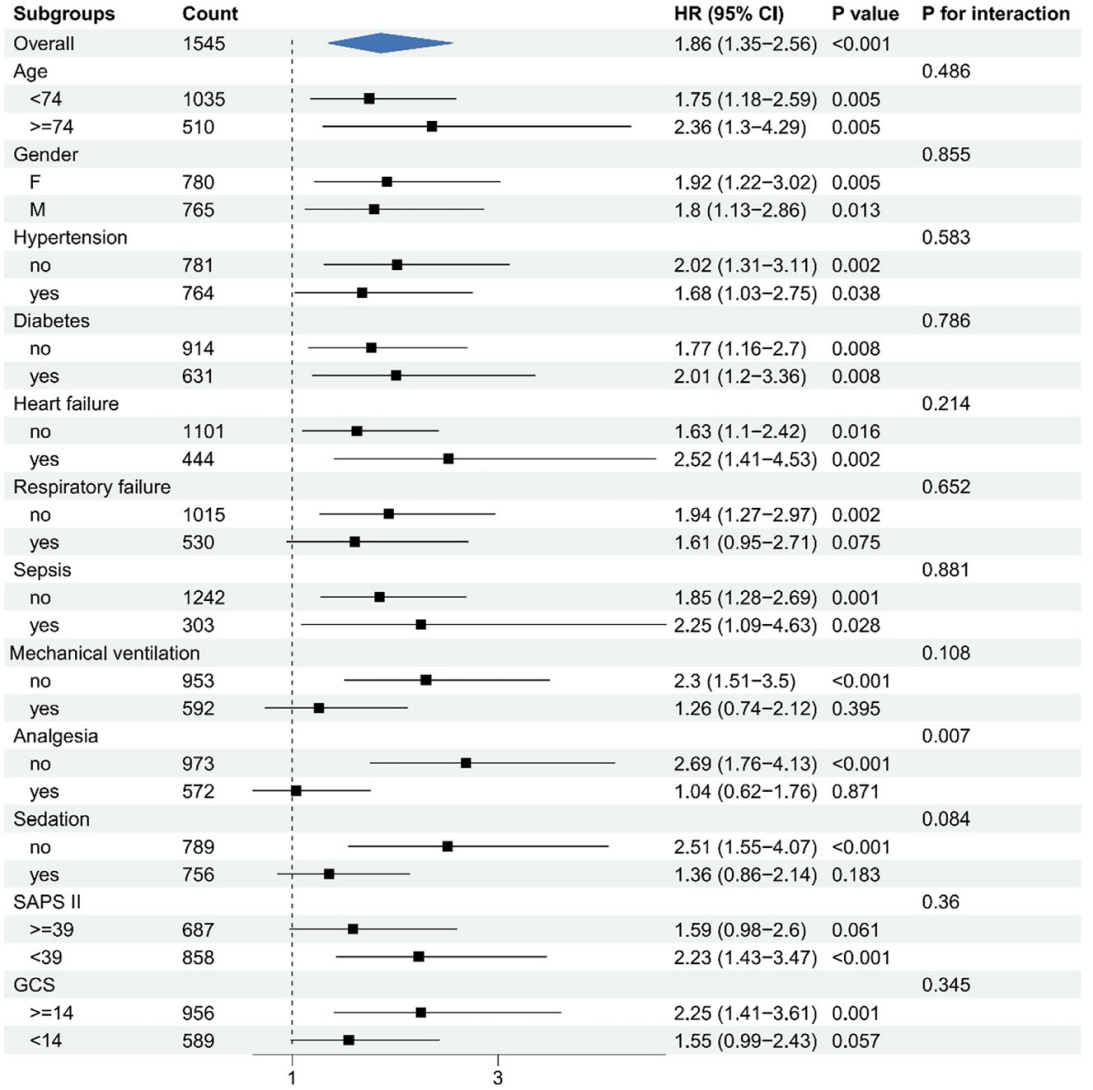
Subgroup analysis of the relationship between pregabalin use and delirium.

**Figure 4:**
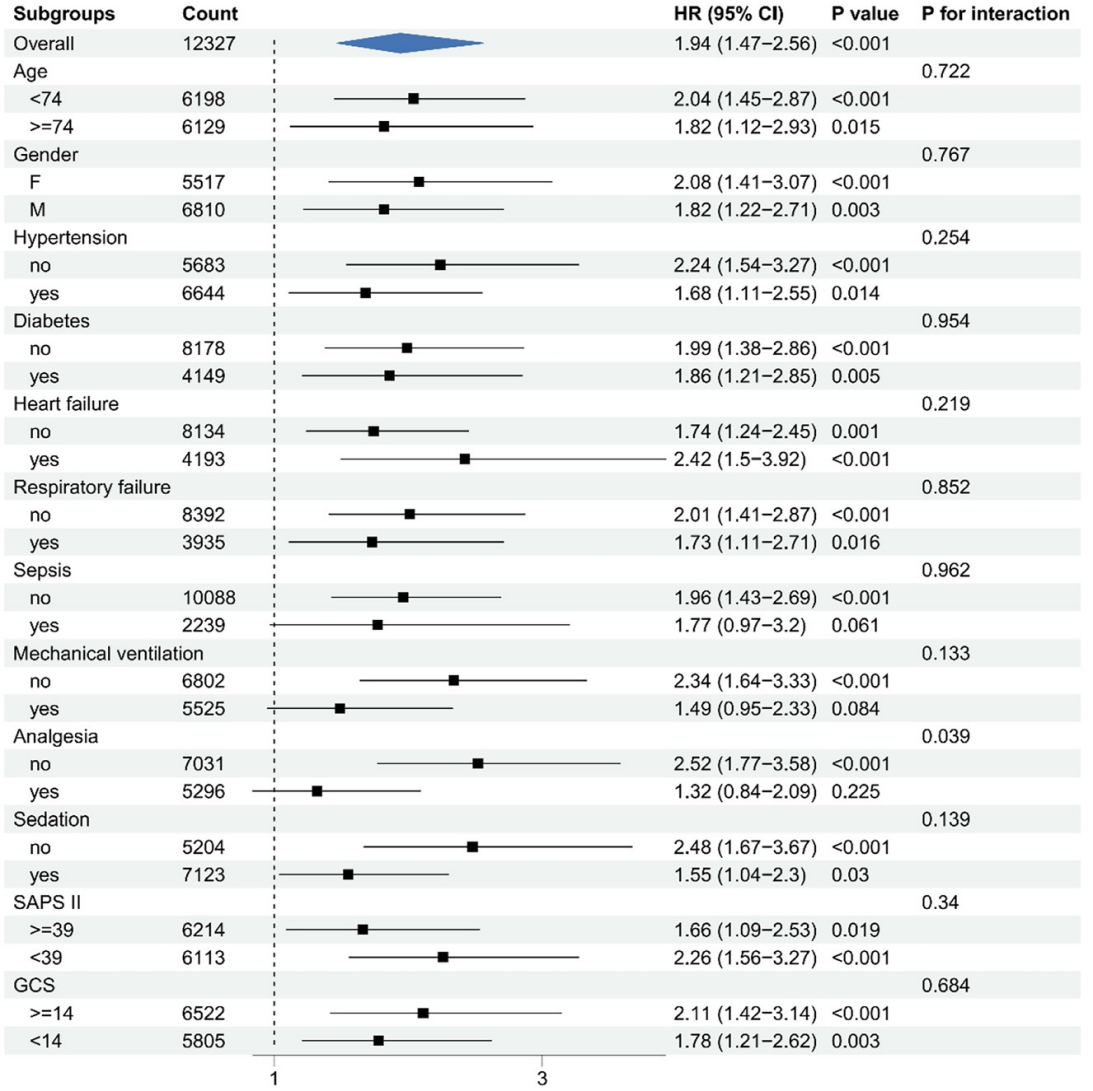
Pre-PSM subgroup analyses adjusted for analgesia.

Risk stratification for the primary outcome was conducted across multiple subgroups, including age, gender, hypertension, diabetes, heart failure, respiratory failure, sepsis, mechanical ventilation, analgesia, sedation, SAPS II, and GCS. Figure 3 illustrates the association between pregabalin use and delirium within each subgroup. No significant interactions were observed for most variables, indicating that the effect of pregabalin on delirium risk was consistent across these subgroups (all p for interaction>0.05). However, a significant interaction was found in the analgesia subgroup (p for interaction <0.05), where pregabalin use was associated with a markedly higher risk of delirium in patients not receiving analgesia (HR:2.69, 95 % CI:1.76–4.13). Similar results were observed in pre-PSM subgroup analyses adjusted for analgesia, further supporting the robustness and consistency of the findings (Figure 4).

## Discussion

This study examined the association between pregabalin use and delirium in elderly ICU patients. Using PSM to control for confounding variables, we found that pregabalin use was significantly associated with an increased risk of delirium in patients aged 60 and older. This association remained consistent across most subgroup analyses. When contextualizing our findings within the broader literature on gabapentinoids, the evidence appears complex. A recent systematic review of RCTs found inconsistent effects of gabapentin on postoperative delirium, showing benefit in spinal surgery but no significant effect in arthroplasty patients, while pregabalin similarly demonstrated no protective effect in elective total hip arthroplasty [26]. Furthermore, a large retrospective study in older colorectal surgery patients revealed that the relationship between gabapentin and delirium risk is highly dose-dependent and influenced by preoperative exposure history [27]. Compared to this mixed evidence for gabapentin, our study demonstrates a more consistent and significant association specifically for pregabalin in elderly ICU patients. This suggests that while gabapentinoids as a class may influence delirium risk, the effect appears more pronounced with pregabalin, potentially due to its higher bioavailability and more potent binding affinity at the α2δ subunit of voltage-gated calcium channels.

Delirium is a common complication in hospitalized older adults, particularly in critical care settings [13]. This acute neurocognitive disorder manifests as disturbances in attention, awareness, cognition, and perceptual-motor function. In elderly patients, somatic symptoms such as urinary incontinence, gait instability, tremors, and speech disturbances are also frequently observed [28]. The underlying pathophysiology involves transient disruption of normal neuronal activity, primarily driven by altered neurotransmission and network dysfunction in response to systemic insults. Delirium pathogenesis is multifactorial, encompassing genetic predisposition, neurotransmitter dysregulation, neuroinflammation, impaired cerebral perfusion, and metabolic disturbances [29], 30]. Advanced age represents a well-established independent risk factor for delirium in hospitalized patients [31]. Age-related cerebral changes – including altered neurotransmitter dynamics, neuronal loss [32], 33], reduced functional specialization of brain regions [34], decreased cerebral blood flow [35], and impaired intracellular signaling [36] – further exacerbate vulnerability to delirium during critical illness.

In high-risk individuals, delirium arises from the brain’s reduced capacity to respond to acute physiological stress. This impairment may involve disrupted brain network connectivity, neuroinflammation, cerebrovascular dysfunction, and altered neurotransmitter synthesis and signaling [37]. Clinically, delirium presents with fluctuating levels of consciousness, arousal, and cognition, reflecting failures in neural integration and processing, often linked to impaired consciousness [38], 39]. Normal consciousness relies on the structural and functional integrity of interconnected brain networks, which can be compromised by aging and neurodegenerative processes [40]. These changes may also impair cerebral perfusion, diminish vascular reactivity, and disrupt BBB integrity, reducing the transport of essential proteins and increasing vulnerability to hypoxia, metabolic stress, and inflammation [13], 41]. At the neurochemical level, delirium is associated with decreased acetylcholine (ACh) availability and excessive dopamine (DA), norepinephrine (NE), and glutamate (GLU) activity, alongside dysregulation of serotonin (5-HT), histamine, and GABA systems [30]. The predominant hypothesis posits that dopaminergic hyperactivity and cholinergic hypoactivity are central to delirium pathogenesis, with downstream effects on GLU and GABA pathways [42]. Drug-induced delirium may result from direct pharmacologic disruption of these neurotransmitter systems. Changes in medication, including initiation, withdrawal, or dose adjustments, can precipitate delirium through neurochemical imbalance [43]. Commonly implicated agents include benzodiazepines, opioids, dihydropyridines, and antihistamines [44]. While inadequate or inappropriate analgesic sedation may contribute, the relationship between pain management, analgesic use, and delirium remains complex and not fully understood.

Pregabalin is approved for the treatment of neuropathic pain and refractory epilepsy [45], 46]. Structurally similar to GABA, pregabalin crosses the BBB efficiently due to its lipophilic modifications [16]. It selectively binds to the α2δ-1 and α2δ-2 subunits of voltage-gated calcium channels, primarily expressed on excitatory and inhibitory neurons, respectively. Notably, presynaptic α2δ-2 subunits promote clustering of postsynaptic GABA receptors, suggesting that pregabalin modulates the excitatory-inhibitory balance within neural circuits [47], 48]. This interaction reduces the presynaptic release of several neurotransmitters – most notably GLU – through modulation of GLU synthesis via branched-chain amino acid transaminase [17], 49], 50]. More broadly, pregabalin diminishes the release of GLU, NE, substance p, ACh and 5-HT [51], 52]. Although case reports have described delirium associated with pregabalin use and withdrawal [21], [22], [23, 53], this study is the first to present clinical evidence linking pregabalin use to increased delirium risk in elderly ICU patients and to explore potential mechanisms. Subgroup analyses further revealed that this association was particularly significant in patients not receiving analgesia. We hypothesize that the absence of analgesia co-administration may reduce neurological confounders, thereby clarifying pregabalin’s independent effect on delirium risk. Notably, our findings prompt a more critical examination of the potential mechanisms underlying pregabalin-associated neuropsychiatric complications. While pregabalin is commonly prescribed for pain and anxiety in critically ill patients, its therapeutic targets may also contribute to delirium risk. The drug’s modulation of excitatory and inhibitory neurotransmission – particularly through α2δ subunit binding – may disrupt delicate neurochemical balances in vulnerable elderly brains. Furthermore, the presence of comorbid pain or anxiety, which often necessitates pregabalin use, may itself exacerbate neurophysiological stress and predispose patients to delirium. This creates a complex clinical scenario where the treatment for certain conditions may inadvertently contribute to neuropsychiatric complications. However, our study lacks detailed data on the onset, duration, and clinical features of delirium episodes during pregabalin therapy. Tomašić et al. proposed that abrupt discontinuation of benzodiazepines, which act as allosteric modulators of GABA_A receptors, leads to reduced neuronal inhibition and heightened excitability – mechanisms that may similarly apply to pregabalin [54]. Additionally, high or fluctuating pregabalin doses may dysregulate neurotransmitter systems involved in delirium, including GABA, GLU, NE, substance p, ACh, and 5-HT. GABAergic dysfunction in key brain regions – such as the hippocampus, prefrontal cortex, limbic system, and subcortical areas – has been implicated in cognitive impairment and may underlie pregabalin-related neuropsychiatric effects [21]. Moreover, compromised BBB integrity in critically ill patients may enhance pregabalin penetration into the CNS, amplifying its psychoactive potential. Given the high prevalence of delirium in ICU settings, identifying modifiable risk factors is crucial. Few clinical studies have addressed the relationship between pregabalin and delirium in elderly populations. Our findings fill this gap and provide novel insights that may inform safer prescribing practices in critical care.

This study has several limitations. First, as a single-center analysis based on the MIMIC-IV database, the findings require validation in larger, multicenter cohorts due to the limited representation of patients treated with pregabalin. Second, data on pregabalin dosage, duration, and its temporal relationship to delirium onset were not recorded, limiting causal inference. Third, potential pharmacodynamic interactions between pregabalin and concomitant medications remain unclear. Although PSM was used to mitigate confounding from baseline variables, unmeasured confounders and selection bias may still influence the results. Further research is needed to clarify pregabalin’s role in delirium. Future prospective studies should address these limitations, particularly by capturing the timing of delirium onset and evaluating the impact of varying pregabalin dosages. Recent advances in artificial intelligence (AI) have shown promise in disease classification, prediction, and diagnosis in clinical medicine [55], [56], [57], [58], [59]. While this study relied on data from a single center to explore preliminary hypotheses, future work integrating heterogeneous data sources and leveraging AI-driven analytics may offer significant improvements in model performance and clinical applicability.

## Conclusions

In conclusion, pregabalin use is associated with an elevated risk of delirium in critically ill patients aged 60 years and older. However, an important limitation is the potential for confounding by indication, as patients prescribed pregabalin may have systematically differed in unmeasured characteristics such as underlying pain conditions, neuropathic disorders, or psychiatric comorbidities that could independently influence delirium risk. Randomized controlled trials are needed to confirm these findings.

## Supplementary Material

Supplementary Material

## References

[1] Hshieh TT, Inouye SK, Oh ES (2020). Delirium in the elderly. Clin Geriatr Med.

[2] Keenan CR, Jain S (2022). Delirium. Med Clin.

[3] Mart MF, Williams RS, Salas B, Pandharipande PP, Ely EW (2021). Prevention and management of delirium in the intensive care unit. Semin Resp Crit Care.

[4] Bellelli G, Brathwaite JS, Mazzola P (2021). Delirium: a marker of vulnerability in older people. Front Aging Neurosci.

[5] Iglseder B, Fruhwald T, Jagsch C (2022). Delirium in geriatric patients. Wien Med Wochenschr.

[6] Stollings JL, Kotfis K, Chanques G, Pun BT, Pandharipande PP, Ely EW (2021). Delirium in critical illness: clinical manifestations, outcomes, and management. Intensive Care Med.

[7] Thom RP, Levy-Carrick NC, Bui M, Silbersweig D (2019). Delirium. Am J Psychiatr.

[8] Alvarez EA, Garrido MA, Tobar EA, Prieto SA, Vergara SO, Briceno CD (2017). Occupational therapy for delirium management in elderly patients without mechanical ventilation in an intensive care unit: a pilot randomized clinical trial. J Crit Care.

[9] Gross AL, Jones RN, Habtemariam DA, Fong TG, Tommet D, Quach L (2012). Delirium and long-term cognitive trajectory among persons with dementia. Arch Intern Med.

[10] Smith PJ, Attix DK, Weldon BC, Greene NH, Monk TG (2009). Executive function and depression as independent risk factors for postoperative delirium. Anesthesiology.

[11] Wilson K, Broadhurst C, Diver M, Jackson M, Mottram P (2005). Plasma insulin growth factor-1 and incident delirium in older people. Int J Geriatr Psychiatr.

[12] Sanford AM, Flaherty JH (2014). Do nutrients play a role in delirium?. Curr Opin Clin Nutr.

[13] Wilson JE, Mart MF, Cunningham C, Shehabi Y, Girard TD, MacLullich A (2020). Delirium. Nat Rev Dis Primers.

[14] Atterton B, Paulino MC, Povoa P, Martin-Loeches I (2020). Sepsis associated delirium. Medicina-Lithuania.

[15] Zhai W, Liu H, Li J, Xin H (2024). Pregabalin-induced rhabdomyolysis: a case series and literature analysis. J Int Med Res.

[16] Rissardo JP, Caprara A (2020). Pregabalin-associated movement disorders: a literature review. Brain Circ.

[17] Azmi S, ElHadd KT, Nelson A, Chapman A, Bowling FL, Perumbalath A (2019). Pregabalin in the management of painful diabetic neuropathy: a narrative review. Diabetes Ther.

[18] Onakpoya IJ, Thomas ET, Lee JJ, Goldacre B, Heneghan CJ (2019). Benefits and harms of pregabalin in the management of neuropathic pain: a rapid review and meta-analysis of randomised clinical trials. BMJ Open.

[19] Huang YH, Pan MH, Yang HI (2023). The association between gabapentin or pregabalin use and the risk of dementia: an analysis of the national health insurance research database in Taiwan. Front Pharmacol.

[20] Quintero GC (2017). Review about gabapentin misuse, interactions, contraindications and side effects. J Exp Pharmacol.

[21] Caliskan AM, Inanli I, Caliskan S, Eren I (2021). Delirium after pregabalin Withdrawal. Alpha Psychiat.

[22] Awasthi H, Vohra A (2023). Delirium following pregabalin discontinuation in an individual with no psychiatric or substance use history. BMJ Case Rep.

[23] Hickey C, Thomas B (2012). Delirium secondary to pregabalin. Gen Hosp Psychiatry.

[24] Noghrehchi F, Stoklosa J, Penev S, Warton DI (2021). Selecting the model for multiple imputation of missing data: just use an IC. Stat Med.

[25] Ely EW, Margolin R, Francis J, May L, Truman B, Dittus R (2001). Evaluation of delirium in critically ill patients: validation of the confusion assessment method for the intensive care unit (CAM-ICU). Crit Care Med.

[26] Gupta A, Joshi P, Bhattacharya G, Lehman M, Funaro M, Tampi DJ (2022). Is there evidence for using anticonvulsants in the prevention and/or treatment of delirium among older adults?. Int Psychogeriatr.

[27] Rajan A, Monteiro JFG, Mujahid N, Vrees M, Schechter S, McNicoll L (2023). Does postoperative gabapentin analgesia add to delirium in older colorectal surgery patients?. Innov Aging.

[28] Saxena S, Lawley D (2009). Delirium in the elderly: a clinical review. Postgrad Med J.

[29] Inouye SK, Westendorp RG, Saczynski JS (2014). Delirium in elderly people. Lancet.

[30] Maldonado JR (2018). Delirium pathophysiology: an updated hypothesis of the etiology of acute brain failure. Int J Geriatr Psychiatr.

[31] Pinho C, Cruz S, Santos A, Abelha FJ (2016). Postoperative delirium: age and low functional reserve as independent risk factors. J Clin Anesth.

[32] Kochunov P, Ramage AE, Lancaster JL, Robin DA, Narayana S, Coyle T (2009). Loss of cerebral white matter structural integrity tracks the gray matter metabolic decline in normal aging. Neuroimage.

[33] Villeda S, Brown-Borg H, Anderson R (2023). Neurobiology of aging: new insights from across the research spectrum. J Gerontol a-Biol.

[34] Goh JO (2011). Functional dedifferentiation and altered connectivity in older adults: neural accounts of cognitive aging. Aging Dis.

[35] Galiano A, Mengual E, Garcia DER, Galdeano I, Vidorreta M, Recio M (2020). Coupling of cerebral blood flow and functional connectivity is decreased in healthy aging. Brain Imaging Behav.

[36] Ungvari Z, Tarantini S, Donato AJ, Galvan V, Csiszar A (2018). Mechanisms of vascular aging. Circ Res.

[37] Maldonado JR (2013). Neuropathogenesis of delirium: review of current etiologic theories and common pathways. Am J Geriatr Psychiatr.

[38] Sanders RD (2011). Hypothesis for the pathophysiology of delirium: role of baseline brain network connectivity and changes in inhibitory tone. Med Hypotheses.

[39] Ferrarelli F, Massimini M, Sarasso S, Casali A, Riedner BA, Angelini G (2010). Breakdown in cortical effective connectivity during midazolam-induced loss of consciousness. P Natl Acad Sci Usa.

[40] Tijms BM, Wink AM, de Haan W, van der Flier WM, Stam CJ, Scheltens P (2013). Alzheimer’s disease: connecting findings from graph theoretical studies of brain networks. Neurobiol Aging.

[41] Sweeney MD, Kisler K, Montagne A, Toga AW, Zlokovic BV (2018). The role of brain vasculature in neurodegenerative disorders. Nat Neurosci.

[42] Gaudreau JD, Gagnon P (2005). Psychotogenic drugs and delirium pathogenesis: the central role of the thalamus. Med Hypotheses.

[43] Maclullich AM, Ferguson KJ, Miller T, de Rooij SE, Cunningham C (2008). Unravelling the pathophysiology of delirium: a focus on the role of aberrant stress responses. J Psychosom Res.

[44] Clegg A, Young JB (2011). Which medications to avoid in people at risk of delirium: a systematic review. Age Ageing.

[45] Tassone DM, Boyce E, Guyer J, Nuzum D (2007). Pregabalin: a novel gamma-aminobutyric acid analogue in the treatment of neuropathic pain, partial-onset seizures, and anxiety disorders. Clin Ther.

[46] Wang Y, Yang H, Shen C, Luo J (2017). Morphine and pregabalin in the treatment of neuropathic pain. Exp Ther Med.

[47] Li Z, Taylor CP, Weber M, Piechan J, Prior F, Bian F (2011). Pregabalin is a potent and selective ligand for alpha(2)delta-1 and alpha(2)delta-2 calcium channel subunits. Eur J Pharmacol.

[48] Alles S, Cain SM, Snutch TP (2020). Pregabalin as a pain therapeutic: beyond calcium channels. Front Cell Neurosci.

[49] Fink K, Dooley DJ, Meder WP, Suman-Chauhan N, Duffy S, Clusmann H (2002). Inhibition of neuronal Ca(2+) influx by gabapentin and pregabalin in the human neocortex. Neuropharmacology.

[50] Hutson SM, Berkich D, Drown P, Xu B, Aschner M, LaNoue KF (1998). Role of branched-chain aminotransferase isoenzymes and gabapentin in neurotransmitter metabolism. J Neurochem.

[51] Dooley DJ, Taylor CP, Donevan S, Feltner D (2007). Ca2+ channel alpha2delta ligands: novel modulators of neurotransmission. Trends Pharmacol Sci.

[52] Brawek B, Loffler M, Dooley DJ, Weyerbrock A, Feuerstein TJ (2008). Differential modulation of K(+)-evoked (3)H-neurotransmitter release from human neocortex by gabapentin and pregabalin. N-S Arch Pharmacol.

[53] Bhatt A (2020). Delirium and pregabalin use. Am J Psychiatr Resid J.

[54] Tomašić L, Kovačić Petrović Z (2023). GABAergic psychoactive substance-induced delirium: narrative literature review. Arch Psychiatr Research: An Int J Psychiatry Relat Sci.

[55] Hafizović L, Čaušević A, Deumić A, Bećirović LS, Pokvić LG, Badnjević A (2021). The use of artificial intelligence in diagnostic medical imaging: systematic literature review. 2021 IEEE 21st International Conference on Bioinformatics and Bioengineering (BIBE).

[56] Badnjević A, Cifrek M, Koruga D (2013). Integrated software suite for diagnosis of respiratory diseases. Eurocon 2013.

[57] Hrvat F, Spahić L, Pokvić LG, Badnjević A (2020). Artificial neural networks for prediction of medical device performance based on conformity assessment data: infusion and perfusor pumps case study. 2020 9th Mediterranean conference on embedded computing (MECO).

[58] Veljović E, Špirtović-Halilović S, Muratović S, Osmanović A, Badnjević A, Gurbeta L (2017). Artificial neural network and docking study in design and synthesis of xanthenes as antimicrobial agents. CMBEBIH 2017: Proceedings of the International Conference on Medical and Biological Engineering 2017.

[59] Begic E, Gurbeta PL, Begic Z, Begic N, Dedic M, Mrsic D (2021). From heart Murmur to echocardiography - congenital heart defects diagnostics using machine-learning algorithms. Psychiatr Danub.

