# Supplementary material for: Association between pregabalin use and delirium in critically ill patients aged 60 and older: a retrospective analysis of the MIMIC-IV database
Source: Open Med (Wars). 2026 Jan 22;21(1):20251354. doi: 10.1515/med-2025-1354 (PMC12917587; doi:10.1515/med-2025-1354)
Supplement: Supplementary file 1 — Supplementary Material [file j_med-2025-1354_suppl_001.docx]

**Supplementary materials**


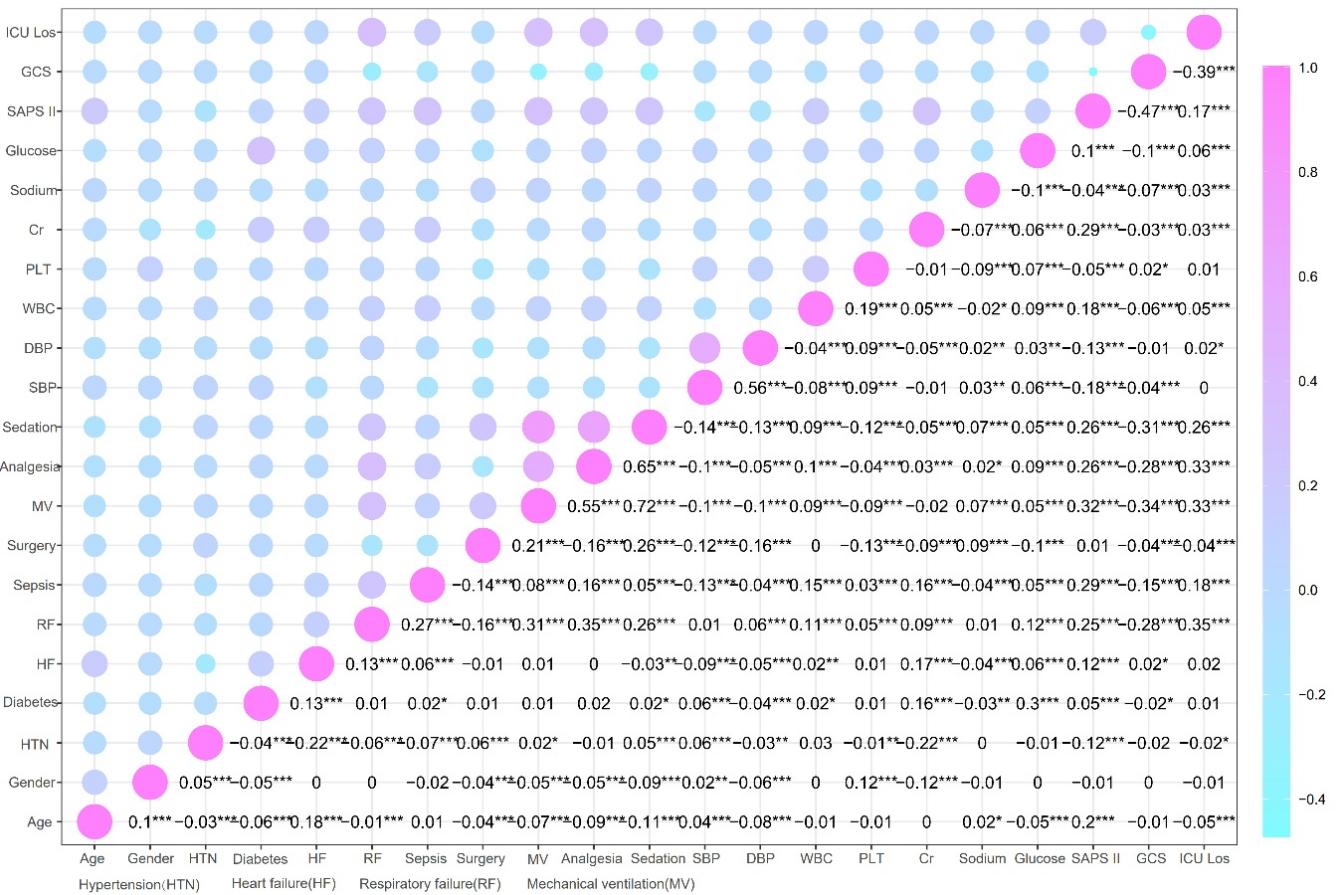


**Figure S1.** Correlation analysis indicated no strong correlation among the covariates.


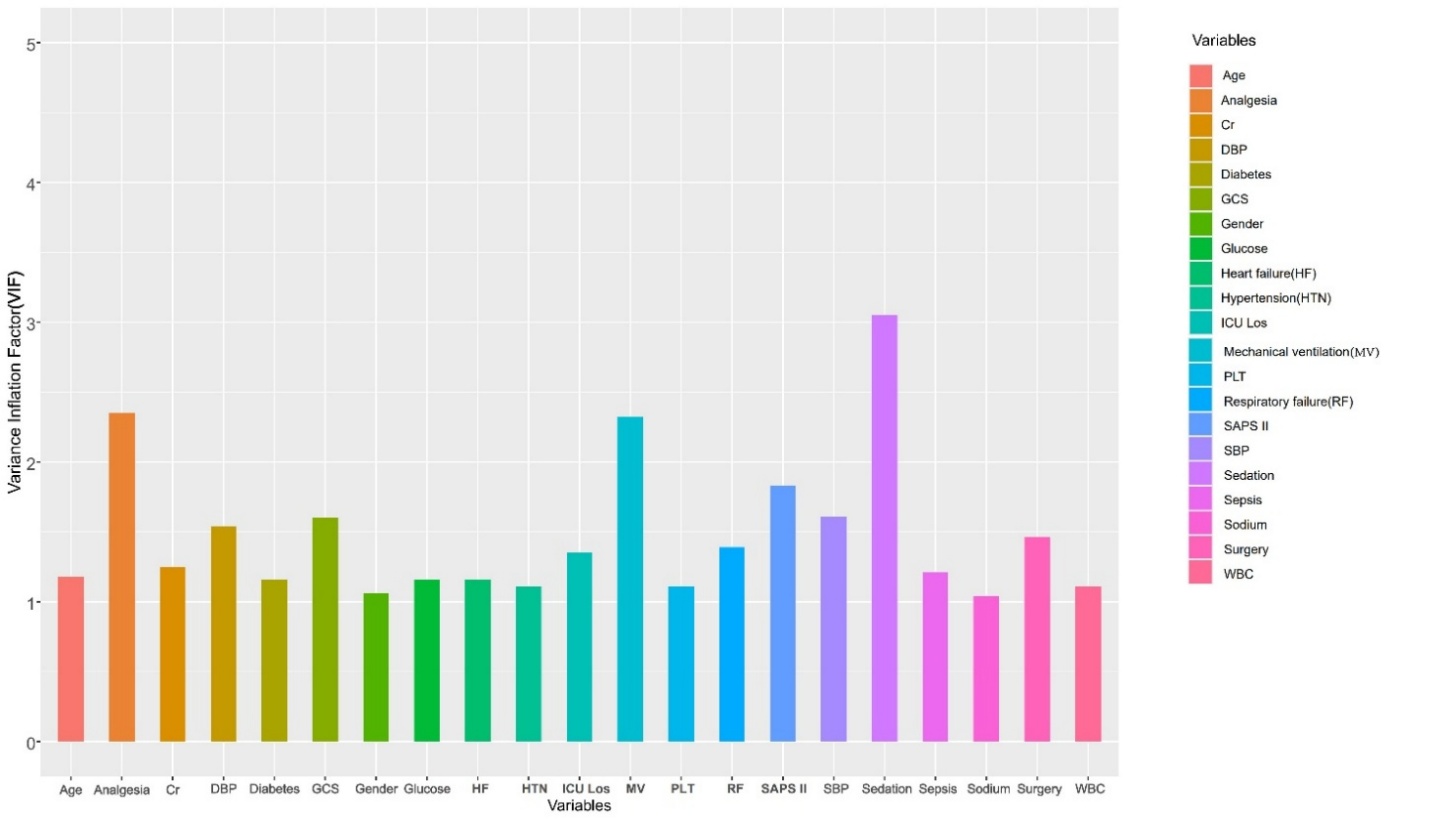


**Figure S2.** Collinearity analysis indicated no high multicollinearity among the covariates.
